# DYNAMIC BALANCE PERFORMANCE WAS ASSOCIATED WITH BETTER COGNITION IN COMMUNITY-DWELLING LOW-INCOME OLDER ADULTS

**DOI:** 10.1093/geroni/igae098.2012

**Published:** 2024-12-31

**Authors:** Ladda Thiamwong, Renata Komalasari

**Affiliations:** University of Central Florida, Orlando, Florida, United States; Tzu Chi Hospital, Jakarta, Indonesia

## Abstract

**Objectives:**

We cross-sectionally examined the relationships of dynamic and static balance performance, and handgrip strength, with cognitive performance in community-dwelling low-income older adults in Orlando, Florida.

**Methods:**

This study was drawn from a sample of 92 older adults (90.2% females, Mage = 72.63, SD±6.82). Dynamic balance was measured by the Short Physical Performance Battery, static balance by BTrackS scores, handgrips by three handgrip trials, and cognitive performance by the Rowland Universal Dementia Assessment Scale.

**Results:**

Across the participants, 71.7% were African American, 62% completed high school, and 57.6% were just enough financially. The multiple regression analysis indicated that the model explained 10.2% of the variance (R2=.319, F (3,88) = 3.33, p =.023). Dynamic balance (B =.075, p-value =.047) was significantly associated with cognitive performance, but static balance (B =-.023, p-value =.081) and handgrip strength were not (B =.002, p-value =.855), accounting for current depressive symptoms (B =.057, p-value =.374). Independent t-test showed that older adults with normal cognition (RUDAS scores ≥23) scored higher in dynamic balance (Mdifference= 27.845, p=.018) than those with possible cognitive impairment (RUDAS scores < 23). Older adults who are cognitively healthy performed better than those with possible cognitive impairment in dynamic balance performance.

**Discussion:**

Simple dynamic balance performance tests, comprising balance, gait speed, and chair stand tests, could serve as a valuable cognitive assessment tool in community-dwelling low-income older adults. Keywords: dynamic balance performance, static balance performance, handgrip strength, cognitive performance, Rowland Universal Dementia Assessment Scale, aging

